# Influence of Post-Processing Techniques on Surface Roughness, Wettability, and Friction of SLM-Manufactured CoCrW Orthodontic Materials

**DOI:** 10.3390/jfb17070315

**Published:** 2026-06-30

**Authors:** Kağan Berk, Aykut Can Önel, Karahan Ocak, Yasemin Tabak, Aisha Gokce Ozbay, Veda Duman Kantarcioglu, Kaan Orhan, Salih Veziroglu, Oral Cenk Aktas, Sinan Şen

**Affiliations:** 1 Department of Orthodontics, University Hospital Schleswig Holstein, Arnold-Heller-Strasse 3, 24105 Kiel, Germany; 2 TUBITAK UME (National Metrology Institute), Kocaeli 41470, Türkiyeyasemin.tabak@tubitak.gov.tr (Y.T.); gokce.ozbay@tubitak.gov.tr (A.G.O.); 3 Department of Criminalistic, Institute of Forensic Sciences, Ankara University, Ankara 06620, Türkiye; 4 Department of Oral and Maxillofacial Radiology, Faculty of Dentistry, Ankara University, Ankara 06560, Türkiye; 5 Chair for Composite Materials, Department of Materials Science, Faculty of Engineering, Kiel University, Kaiserstraße 2, 24143 Kiel, Germany

**Keywords:** CoCrW alloys, selective laser melting (SLM): orthodontic materials, surface roughness, additive manufacturing

## Abstract

This study investigates the effects of post-processing on the surface roughness, wettability, and frictional behavior of selective laser-melted (SLM) cobalt–chromium–tungsten (CoCrW) alloys for orthodontic use. The SLM-CoCrW specimens were tested in as-manufactured, mechanically polished, and electropolished states. Surface characterization via stylus profilometry and atomic force microscopy (AFM) showed that both polishing methods reduced macro- and micro-scale roughness, with electropolishing producing the smoothest, most uniform topography. Static water contact angle (WCA) measurements revealed that mechanical polishing provided an optimal balance of roughness and hydrophilicity, resulting in the lowest friction, while ultrasmooth electropolished surfaces exhibited slightly higher friction due to increased hydrophobicity and a uniform Cr-rich oxide layer confirmed by X-ray photoelectron spectroscopy (XPS). XPS also indicated that electropolishing generated a homogenous chromium oxide passive film, whereas mechanical polishing left a chemically heterogeneous surface with exposed metallic sites. Importantly, performance is not governed solely by surface roughness; surface chemistry is equally critical, and both must be considered together—along with wettability and tribological behavior—to achieve optimal functional outcomes. From a clinical perspective, optimization of surface roughness and surface chemistry may result in decreased frictional resistance, improved sliding mechanics, and enhanced long-term performance of additively manufactured orthodontic components; however, the present study was restricted to in vitro characterization under simplified laboratory conditions, and further investigations involving artificial saliva, long-term aging, wear and clinical simulations are necessary to validate the translational relevance of these findings.

## 1. Introduction

An important factor in effective orthodontic treatment is the materials used for the appliances, which need to offer not only structural integrity and biocompatibility but also optimal surface properties [1]. These properties significantly influence the interaction between orthodontic devices and biological tissues, ultimately affecting treatment outcomes and patient comfort [2]. Surface properties play a pivotal role in orthodontics, influencing the performance, longevity, and clinical outcomes of various orthodontic materials [3,4]. From archwires and brackets to removable appliances, the surface directly affects frictional resistance, microbial adhesion, and overall treatment efficiency [5,6]. Understanding the correlation between the surface and these factors is essential for optimizing orthodontic care and improving patient outcomes.

Surface roughness is especially important for orthodontic archwires and brackets, as they form the basis for fixed orthodontic appliances [7]. Thereby, it has been shown that higher surface roughness of archwires has a significant effect on the frictional resistance in the archwire–bracket system and, hence, the efficiency of tooth movement during sliding mechanics [8]. In metallic orthodontic systems, it has been shown that the surface roughness and frictional resistance vary substantially based on the type of the alloy, bracket design, and surface finishing method [9]. Passive self-ligating stainless steel orthodontic brackets are less prone to surface roughness at the nanoscale, and frictional behavior has been indicated as lower than that of ceramic self-ligating brackets in general [10,11]. These results confirm that the frictional behavior is not only determined by the broad material class (metals, ceramics, polymers, etc.) but also depends significantly on intrinsic material characteristics, surface chemistry, oxide layer properties, and micro-/nano-scaled topographical conditions [12]. Such selection and engineering of metallic orthodontic materials is crucial to minimize friction, improve sliding mechanics, and improve orthodontic treatment efficiency [13].

Surface roughness influences not only the resistance to friction but also the biocompatibility and corrosion characteristics of orthodontic materials [14]. Rougher surfaces increase the interaction area, promoting microbial attachment and biofilm formation, especially in difficult oral cavity conditions. This issue has sparked considerable interest in metallic orthodontic alloys, including in surface and near-surface defects such as microcracks, porosity, and multi-scale roughness that can act as advantageous sites for microbial adhesion and localized corrosion, especially in the face of variable oral chemical conditions [15]. Previous studies showed in vitro that exposure of stainless steel and nickel–titanium (NiTi) orthodontic components to fluoride-containing and chlorhexidine mouthwashes leads to increased surface roughness and altered surface morphology, influencing the frictional properties, corrosion resistance, and ion release of metallic appliances [16]. These results suggest that chemical interactions with the oral environment, not solely physical ones, can seriously dictate the structural integrity and functional properties of metallic orthodontic systems. SLM commonly has a layer-by-layer additive manufacturing process resulting in surfaces appearing more rough than normal surfaces. The roughness characteristic of SLM materials is affected by a number of factors, including laser parameters, powder features, and cooling condition during solidification [17]. High long-range roughness (macro-scale) may also occur concurrently with excessive short-range (micro-/nanoscale) roughness in additively manufactured CrCo or Ti-based alloys [18]. Therefore, a multi-scale roughness assessment is vital to accurately predict frictional behavior, biological response, and mechanical reliability in modern orthodontic systems.

Of the orthodontic materials employed, cobalt–chromium–tungsten (CoCrW) alloys manufactured by SLM have attracted increasing attention owing to their favorable mechanical properties and proven biocompatibility [19]. SLM is an advanced additive manufacturing process capable of the precise creation of complex and patient-specific geometries, which is of high importance for customized orthodontic devices. The intrinsic properties of SLM-CoCrW materials, including a high strength-to-weight ratio, excellent wear and corrosion resistance, and structural stability, make them attractive for orthodontic applications [20]. Nevertheless, the SLM technology produces surfaces with high roughness, partially melted particles, and anisotropic topographical features, which adversely affect frictional behavior and clinical performance. Previous studies have shown that the surface quality and functional behavior of additively manufactured CoCr-based dental alloys are strongly influenced by post-processing conditions due to the inherently rough surface morphology generated during the SLM process. Therefore, surface finishing treatments are considered essential for improving the functional performance and clinical applicability of SLM-fabricated orthodontic materials. In this respect, post-processing is an indispensable task for SLM-CoCrW orthodontic components. Surface treatments such as mechanical polishing, electropolishing, plasma/ion-assisted polishing, and chemical or electrochemical finishing are major factors in tailoring surface topography, surface chemistry, and wettability, which directly influence friction, wear mechanisms, and archwire–bracket interactions.

This study comparatively evaluates the effects of mechanical polishing and electropolishing on the surface roughness, wettability, frictional behavior, and surface chemistry of SLM-CoCrW orthodontic alloys. Multiscale surface characterization was conducted using profilometry, atomic force microscopy (AFM), water contact angle (WCA), friction measurements, and X-ray photoelectron spectroscopy (XPS). The study aims to elucidate the combined influence of surface topography and surface chemistry on the functional performance of additively manufactured orthodontic materials.

## 2. Materials and Methods

### 2.1. Selective Laser Melting

Square-shaped plates (8 mm × 8 mm × 2 mm) were fabricated by SLM using a commercial dental CoCrW alloy (Remanium Star CL; Dentaurum GmbH & Co. KG, Ispringen, Germany). The chemical composition of the alloy, as provided by the manufacturer, consisted of 60.5 wt% Co, 28.0 wt% Cr, 9.0 wt% W, 1.5 wt% Si, and <1 wt% Mn, N, Nb, and Fe. The average grain size of the fabricated specimens ranged between 10 and 30 μm. The specimens were manufactured and postprocessed by CADdent^®^ GmbH (Augsburg, Germany) under the charge/lot number 546800, 538486. Postprocessing was performed using mechanical and electropolishing methods routinely applied in dental practice; specific manufacturing and processing parameters are proprietary and therefore not disclosed.

### 2.2. Roughness and Tribological Analysis

Two kinds of roughness (macro and micro) have been evaluated. Long-range (macro) surface roughness of the as-built and post-processed specimens was measured using a contact stylus profilometer (DektakXT, Bruker, Tucson, AZ, USA) under identical experimental conditions. The system operates based on a contact tracing principle, where a diamond stylus scans the surface under a controlled normal load, and vertical displacements caused by surface height variations are recorded to generate path–height profiles. Measurements were performed under identical load and scan-speed conditions for all specimens, with a fixed evaluation length of 2 mm to ensure comparability.

Short-range surface roughness (micro) and nanoscale surface features were characterized by atomic force microscopy (AFM, Park Systems GmbH, XE7, Goettingen, Germany) deployed under identical conditions for all specimens. AFM measurements were performed over a scan area of 45 μm × 45 μm, enabling detailed evaluation of local height variations and fine surface asperities. The combined use of stylus profilometry and AFM provided a multiscale characterization of surface roughness, capturing both long-wavelength surface irregularities and short-wavelength nanoscale features of the investigated specimens.

To investigate the intrinsic frictional behavior of the differently treated SLM-CoCrW surfaces at the microscale, a nano-tribological approach based on AFM was employed. A spherical glass probe with a defined radius was selected to ensure controlled and reproducible contact geometry. During the measurements, the probe was laterally scanned across the surface under controlled normal loads. The normal force was systematically varied (0–20 µN) to evaluate load-dependent frictional behavior. Lateral deflection signals were simultaneously recorded during trace and retrace cycles, and friction forces were calculated from the half-width of the corresponding friction loops. This method allows direct quantification of nanoscale friction while reducing systematic offsets and topography-related artifacts.

All experiments were performed under identical environmental conditions to ensure reliable comparison between the as-manufactured, mechanically polished, and electropolished states. For each surface condition, three independent specimens were analyzed. Given the low variability typically associated with physicochemical surface measurements and the large effect sizes observed between groups, the selected sample size provided sufficient statistical power to detect differences among the investigated surface conditions. Five measurements were acquired from different locations on each specimen to account for local surface variations. The five measurements obtained from each specimen were averaged to generate a specimen-level value. Data are presented as mean ± standard deviation (SD) of the three independent specimens. Statistical comparisons among groups were performed using one-way ANOVA followed by Tukey’s post hoc test, with a significance level of *p* < 0.05.

The above-mentioned analyses were confined to controlled laboratory conditions and did not take into account saliva-mediated lubrication, long-term aging, wear, and other clinically relevant oral environmental factors.

### 2.3. Wettability Analysis

WCA of the as-manufactured and post-processed specimens was measured at room temperature (20–25 °C) using a drop shape analysis system (Krüss GmbH, Hamburg, Germany). During the measurements, a camera positioned above the sample stage continuously monitored the droplet profile, and the contact angle was determined using the system’s image analysis software. A microcapillary with an inner diameter of 5 μm was used to dispense deionized (DI) water droplets onto the sample surface. For each experimental group, three independent specimens were analyzed. Five WCA measurements were acquired from different locations on each specimen and averaged to obtain a specimen-level value. Statistical analysis was performed as described above using specimen-level means, and results are presented as mean ± SD.

### 2.4. Surface Chemistry Analysis

The surface chemical composition of the as-manufactured, mechanically polished, and electropolished SLM-CoCrW specimens was analyzed by X-ray photoelectron spectroscopy (XPS, Phoibos 100, SPECS GmbH, Berlin, Germany) using an ultra-high-vacuum (UHV) system equipped with an Al Kα X-ray source operated at 300 W. Survey spectra were acquired in constant analyzer energy mode with three iterations at a pass energy of 200 eV. High-resolution spectra were recorded with 20 iterations at a pass energy of 50 eV to enable detailed chemical state analysis. The acquired XPS spectra were processed using CasaXPS software (version 2.3.23). A Shirley background was applied for background subtraction, and charge compensation was performed by referencing the C 1 s peak to a binding energy of 284.8 eV. All other core-level spectra were corrected accordingly.

## 3. Results and Discussion

**Figure 1:**
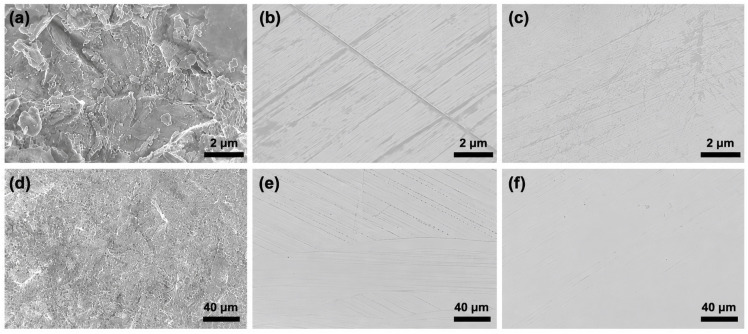
SEM micrographs of SLM-CoCrW specimens. (**a**,**d**) as-manufactured, (**b**,**e**) mechanically polished, and (**c**,**f**) electropolished surfaces. Micrographs (**a**–**c**) represent low-magnification images, while panels (**d**–**f**) show the corresponding higher-magnification images of the same specimens.

SEM images of SLM-CoCrW specimens (Figure 1) provide low- and high-magnification views that reflect the progressive evolution of surface morphology induced by different post-processing conditions. The surface of as-manufactured components presents marked roughness, partially fused and sintered powder particles, balling-related features, and clearly visible melt-pool boundaries, leading to a highly heterogeneous topography with sharp asperities and micro-cavities (Figure 1a,d). These surface properties are consistent with layer-wise fabrication and incomplete powder consolidation typical of SLM processes and are anticipated to facilitate mechanical interlocking and heightened friction during sliding contact [20].

It is illustrated that the surface irregularities decrease substantially after mechanical polishing (Figure 1b,e). These large powder agglomerates and sharp asperities are successfully removed from the surface, producing smooth areas with shallow grooves and directional polishing marks that remain discernible at higher magnification. The electropolished surface, on the other hand, has a markedly uniform and featureless morphology at both magnification levels (Figure 1e,f). Surface asperities, micro-notches, and polishing-induced grooves are largely eliminated, leading to a smooth, continuous surface with minimal topographical contrast. The absence of sharp features and the homogenization of surface relief suggest effective electrochemical material removal, particularly at microscale protrusions.

SLM commonly has a layer-by-layer additive manufacturing process resulting in surfaces appearing more rough than normal surfaces. The roughness characteristic of SLM materials is affected by a number of factors, including laser parameters, powder features, and cooling conditions during solidification [17]. High long-range roughness (macro-scale) may also occur concurrently with excessive short-range (micro-/nanoscale) roughness in advanced orthodontic materials, especially additively manufactured CrCo or Ti-based alloys [18]. Therefore, a multi-scale roughness assessment is vital to accurately predict frictional behavior, biological response, and mechanical reliability in modern orthodontic systems.

**Figure 2:**
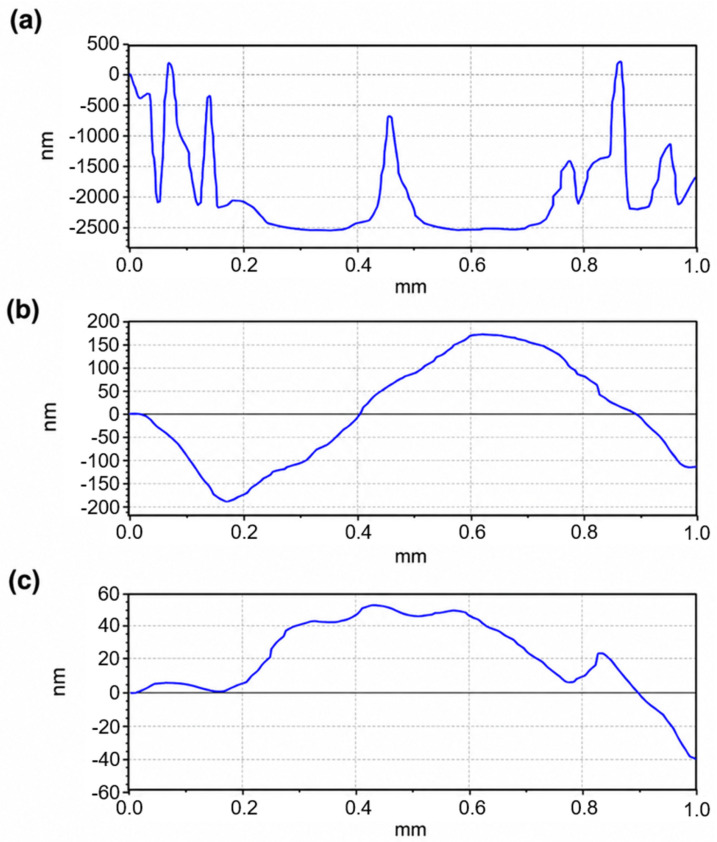
2D profilometer line profiles showing surface height variations of (**a**) as-manufactured, (**b**) mechanically polished and (**c**) electropolished SLM-CoCrW specimens.

A mechanical stylus profilometer was employed to quantitatively assess the long-range surface roughness of the prepared specimens (Figure 2). The mentioned method is particularly effective for capturing long-wavelength surface irregularities and manufacturing-induced topographical features, thereby complementing the nanoscale roughness analysis obtained from AFM and enabling multiscale surface characterization.

The as-manufactured surface is characterized by pronounced peak-to-valley variations and abrupt height transitions, reflecting the presence of partially fused powder particles and melt-pool-related irregularities inherent to the additive manufacturing process (Figure 2a). Such sharp asperities indicate a highly heterogeneous and peak-dominated surface structure. With mechanical polishing, these aggressive surface features are markedly reduced (Figure 2b). The resulting profiles appear smoother and more continuous, primarily composed of gentle, long-wavelength undulations rather than abrupt local peaks. Although minor surface variations remain, the sharp asperity-driven character of the as-built condition is clearly diminished. The electropolished surface exhibits an even more homogenized topography (Figure 2c). The line profile is largely governed by a gradual form component, with minimal evidence of localized protrusions. This behavior suggests effective preferential removal of microscale asperities during electrochemical treatment, leading to a more uniform and stable load-bearing interface. Overall, the profilometry results indicate a clear morphological transition from a rough, peak-dominated surface in the as-manufactured state to a progressively smoother and more topographically homogenized surface after post-processing.

In general, the macro roughness component reflects manufacturing-induced form errors, wire bending marks, bracket slot irregularities, and layer-wise stair-stepping effects in additive manufacturing [21]. Clinically, it is reported that long-range roughness primarily governs mechanical engagement between the archwire and bracket slot, influencing torque expression, force transmission, and load predictability [22]. Macro-scale irregularities can introduce localized stress concentrations, increasing the risk of fatigue damage and mechanical instability, even when micro-scale surface finish appears smooth [23].

**Figure 3:**
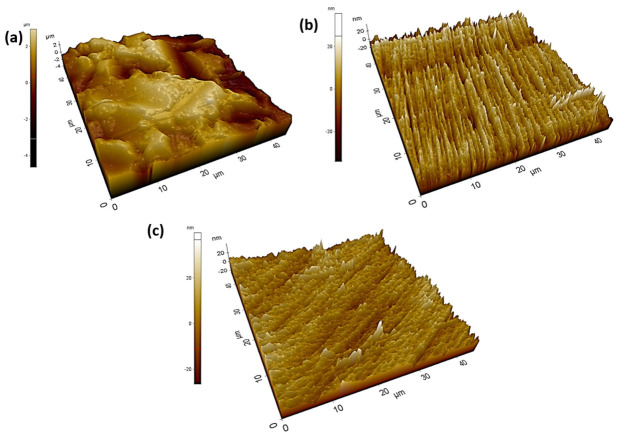
3D AFM-topographical maps of (**a**) as-manufactured, (**b**) mechanically polished and (**c**) electropolished SLM-CoCrW specimens.

**Figure 4:**
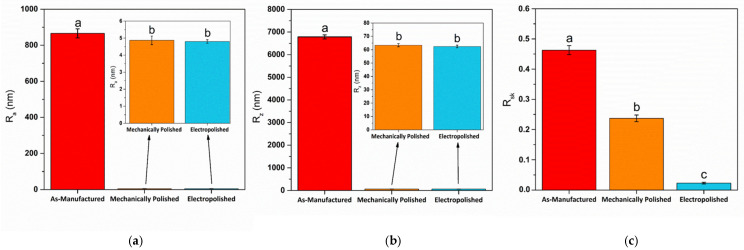
Surface roughness parameters of as-built, mechanically polished and electropolished SLM-CoCrW specimens: (**a**) R_a_, (**b**) R_z_, and (**c**) R_sk_. Data are presented as mean ± SD (n = 3). Different lowercase letters indicate statistically significant differences among groups; groups sharing the same letter are not significantly different according to one-way ANOVA followed by Tukey’s post hoc test (*p* < 0.05).

AFM was used to characterize, in addition to profilometry, short-range surface roughness and surface features with characteristic dimensions from nanometers to a few micrometers. Three-dimensional topographical maps with nanometer-level resolution have been recorded and developed to make a comprehensive comparison (Figure 3). One-way ANOVA revealed statistically significant differences among the three investigated surface groups. Tukey’s post hoc analysis demonstrated that the as-built specimens exhibited significantly higher roughness values than both mechanically polished and electropolished specimens (*p* < 0.001 for both comparisons). No statistically significant difference was observed between the mechanically polished and electropolished groups (*p* = 1.000). In the as-manufactured condition, the specimens exhibited pronounced surface irregularities, reflected by high roughness values, with the average roughness (R_a_) of 866 ± 25.1 nm and the peak-to-valley roughness (R_z_) reaching similarly high values of 6785 ± 88.5 nm (Figure 4). These elevated roughness parameters are typical of SLM-fabricated metallic components and originate from partially fused powder particles, balling phenomena, and layer-wise solidification effects. After post-processing, a significant improvement in surface quality was observed. Surface roughness was significantly reduced following both mechanical polishing and electropolishing. The R_a_ values decreased to 4.87 ± 0.25 nm and 4.80 ± 0.10 nm for the mechanically polished and electropolished specimens, respectively. Similarly, the corresponding R_z_ values were 63.4 ± 1.16 nm and 62.4 ± 1.02 nm, indicating a substantial reduction in surface irregularities compared with the as-manufactured condition. This corresponds to a reduction of more than two orders of magnitude in both roughness parameters compared to the as-manufactured state, indicating the highly effective removal of surface asperities and extreme height variations. The simultaneous reduction in R_a_ and R_z_ demonstrates not only surface smoothing but also the elimination of sharp peaks and deep valleys.

A gradual and significant decrease in surface skewness (R_sk_) appeared with respect to the surface conditions, suggesting that a systematic transformation of the load-bearing surface morphology is possible under the conditions of post-processing (Figure 4). The R_sk_ value in the as-manufactured SLM-CoCrW specimen was quite high (0.463 ± 0.015), suggesting that the surface profile is dominated by prominent asperity peaks of partially fused powder particles, layer-wise solidification features, and melt-pool-related inhomogeneities. Such a positive skewness of this magnitude reflects that of additively manufactured metallic surfaces, as the topography is peak dominated at first, where contact is controlled by a small number of high asperities. In addition, the surface features are known to enhance local contact stresses and favor plowing-dominated frictional forces. The R_sk_ value after mechanical polishing decreased to 0.237 ± 0.011, which shows the direction of the trend towards a more equal height distribution and a reduced dominance of surface peaks. The decrease indicates well that mechanical polishing removes sharp asperities and truncates high peaks, yet the remaining positive skew is indicative of residual protrusions and deformation features beneath the surface in the load-bearing region. Therefore, while the mechanical polishing significantly increases uniformity of the surface over as-manufactured, the surface remains to have peak-biased contact behavior, which can still affect friction and wear responses when sliding. In contrast, the electropolished specimen exhibited a dramatically reduced R_sk_ value of 0.023 ± 0.003, corresponding to an overall decrease of more than one order of magnitude compared to the as-manufactured condition. This pronounced reduction demonstrates that electropolishing is highly effective in homogenizing the surface by preferentially dissolving microscopic asperities and minimizing height variations within the core roughness region. The near-elimination of the core roughness depth indicates a transition toward a highly uniform and stable load-bearing surface.

When considered together with the substantial reductions in R_a_ and R_z_, the progressive decrease in R_sk_ demonstrates the enhanced ability of electropolishing to achieve the desired surface topography. These modifications are important in a number of related orthodontic and biomedical situations, because lower core roughness is strongly correlated with lower friction coefficients, higher wear resistance, better wettability control, and more predictable biological interactions.

Short-range roughness refers to the surface texture variations that occur over short distances, typically within the micrometer range [24]. The elevated short-range roughness commonly observed in SLM-manufactured metallic components originates from layer-wise solidification, partially fused powder particles, and process-dependent factors such as laser parameters and powder characteristics, which collectively govern the final surface topography [25]. From a tribological point of view, short-range roughness directly influences the actual area of contact at the bracket–archwire interface to influence the resistance to friction through sliding mechanics. Higher nano-roughness increases the interlocking of asperities and destabilization of lubrication films, increasing the friction coefficient [26]. Biologically, micro- and nano-scale asperities mediate the penetration of proteins and bacterial adhesion that is critical for plaque accumulation in the mouth and in the gingival cavity [27]. Short-range roughness is also closely related to surface chemistry and wettability, which adjust saliva-mediated lubrication and corrosion. Surface finishing technologies, like electropolishing or thin-film coatings, have efficient benefits for short-range roughness and friction without significant influence on macroscale geometry.

The growing interest in SLM-fabricated CoCr-based alloys is also associated with the capability of additive manufacturing to produce highly complex and patient-specific geometries while maintaining adequate mechanical performance and dimensional accuracy [28]. Although short- and long-range roughness are often evaluated separately, optimal orthodontic performance requires their combined control, as smoother surfaces generally reduce friction and wear. Frictional force arises between two bodies in contact, such as the bracket and archwire (when there is relative motion or even the potential for motion) primarily because the contacting surfaces are not perfectly smooth and exhibit inherent asperities [26].

**Figure 5:**
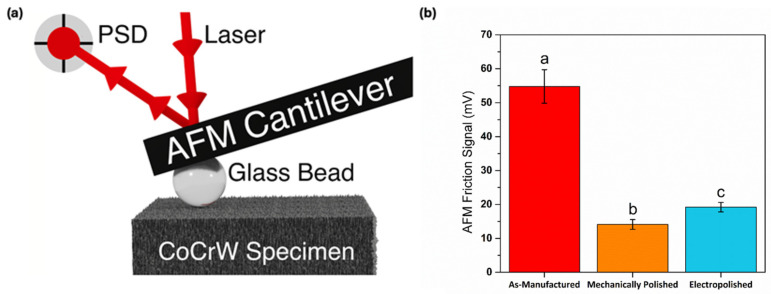
(**a**) Schematic depiction of AFM-based lateral force measurement using spherical probe and (**b**) the friction behavior (as measured by the difference between the forward and backward lateral force signals) of as-manufactured, mechanically polished, and electropolished SLM-CoCrW specimens. Data are presented as mean ± SD based on 3 independent specimens per group. Statistical differences were determined using one-way ANOVA followed by Tukey’s post hoc test). Different lowercase letters indicate statistically significant differences among groups; groups sharing the same letter are not significantly different according to one-way ANOVA followed by Tukey’s post hoc test (*p* < 0.05).

To investigate the mentioned frictional behavior, AFM measurements were conducted using a spherical glass probe with a defined radius, ensuring a controlled and reproducible contact geometry consistent with Hertzian contact mechanics (as depicted in Figure 5a). Unlike conventional macro-scale tribometers, AFM-based lateral force measurements enable highly localized analysis, where nanoscale roughness, surface chemistry, and asperities directly govern interfacial interactions.

**Figure 6:**
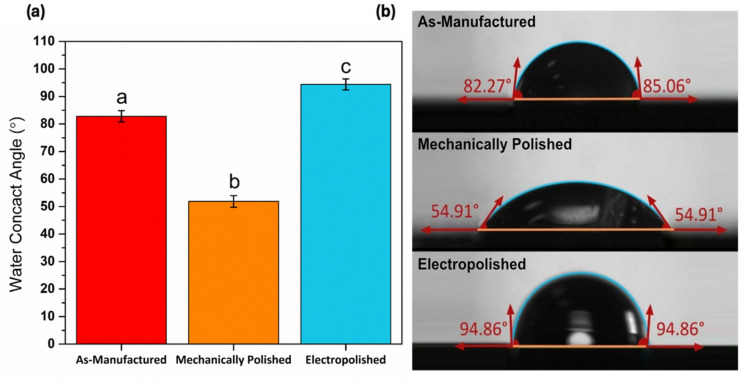
(**a**) WCA analysis and (**b**) corresponding sessile droplet profiles of as-manufactured, mechanically polished, and electropolished SLM-CoCrW specimens. Data are presented as mean ± SD (n = 3). Different lowercase letters indicate statistically significant differences among groups; groups sharing the same letter are not significantly different according to one-way ANOVA followed by Tukey’s post hoc test (*p* < 0.05).

During measurements, the normal load was systematically varied (0–20 µN), and lateral friction forces were extracted from trace–retrace friction loops (Figure 6b). Multiple measurements at different areas ensured statistical reliability. Additionally, the spherical probe reduced tip wear effects, enabling consistent and reliable comparison of frictional behavior across different surface conditions. The friction measurements revealed a clear reduction in the lateral friction signal with surface post-processing. The as-manufactured SLM-CoCrW surfaces exhibited the highest friction signal (54.8 ± 4.92 mV), which can be attributed to their high surface roughness, pronounced asperity heights, and high surface skewness, leading to significant mechanical interlocking at the sliding interface. Mechanical polishing resulted in the lowest friction signal, with an average value of approximately 14.1 ± 1.43. This pronounced reduction is associated with the effective removal of sharp asperities and a substantial decrease in the surface skewness depth, producing a stable and homogeneous load-bearing surface. In addition, the mechanically polished surfaces exhibited the lowest WCA, indicating enhanced hydrophilicity, which may further reduce adhesion forces at the tip–surface interface and contribute to the minimized frictional response.

The electropolished specimen showed slightly higher friction signals (19.2 ± 1.39 mV) compared to mechanically polished surfaces, despite exhibiting the lowest overall roughness values. This suggests that friction is not governed solely by surface roughness. Instead, surface energy and chemistry-dominated interfacial interactions may play an important role in adhesive forces under dry sliding conditions, partially offsetting the benefits of an ultrasmooth surface topography [26]. The wettability of surfaces also plays an important role in friction for orthodontic applications where sliding occurs under wet conditions [29]. Surfaces with higher wettability (lower WCA) promote the formation of stable saliva or water films, which can act as boundary or mixed lubricants and reduce direct asperity contact, thereby lowering frictional resistance. In contrast, poorly wetting, more hydrophobic surfaces tend to disrupt lubricant film continuity, increasing adhesive interactions and the real area of contact, which results in higher friction.

We conducted WCA analysis using a semi-automated goniometer to evaluate the surface wettability of the specimens (Figure 6a,b). As-manufactured surfaces exhibited relatively high WCA, indicating hydrophobic behavior (Figure 6b). A pronounced decrease in WCA was observed after mechanical polishing, resulting in the most hydrophilic surface condition (Figure 6b). Conversely, electropolished surfaces showed an increase in WCA despite exhibiting the lowest roughness values (Figure 6b).

The as-manufactured SLM-CoCrW was characterized by high roughness and heterogeneous asperity distributions, which promote the entrapment of air pockets beneath the liquid droplet [30]. As proposed in the Cassie–Baxter wetting model, such air entrapment can lead to an increase in apparent WCA, resulting in less wettability despite the metallic nature of the surface. Mechanical polishing massively minimizes roughness of the surfaces and homogenizes the asperity distribution and thus effectively suppresses the air entrapment and facilitates a transition to a Wenzel-dominated wetting regime. Consequently, mechanically polished surfaces are characterized by the lowest WCA while being the most hydrophilic. Even when compared to the as-manufactured and mechanically polished specimens, the electropolished SLM-CoCrW specimen showed the lower roughness values but the higher WCA. The results of this study suggest that surface wettability of specimens is affected by more than just topographical roughness but is also susceptible to the impact of chemical interactions on the surface. Electropolishing can create a smoother surface with preferential anodic dissolution of surface asperities but also significantly changes the chemical composition and oxide structure near the surface [30]. Electropolishing promotes the formation of a chromium-rich passive oxide film while simultaneously reducing surface roughness and topographical heterogeneity. Although Cr oxide surfaces are intrinsically hydrophilic, the combined effects of surface smoothing, elimination of high-energy defect sites, and modification of the passive oxide chemistry can reduce the apparent surface free energy, resulting in increased WCA and lower wettability compared with mechanically polished surfaces [30].

In contrast, coarsely polished surfaces may be rougher compared to electropolished surfaces but keep more heterogeneous surface chemistry or higher surface energy because of mechanically induced defects, residual stresses, or exposed metallic sites that result in lower WCA with less uniform surface chemistry. Even though the roughness remains high in as-manufactured SLM surfaces, these can demonstrate relatively lower WCA due to capillary effects and high effective surface area, which favors liquid spreading. Thus, the increase to a maximum WCA observed for the electropolished SLM-CoCrW specimens signifies a shift from roughness-controlled wetting toward a chemistry-dominated wetting behavior, where surface composition and passive oxide characteristics play a more decisive role than surface topography. The results are consistent with the fact that attaining an ultrasmooth surface does not provide better wettability, indicating a major difference in the interaction performance. The combined effects of surface roughness and wettability influence friction, protein adsorption, and biological responses in orthodontic and biomedical applications and should therefore be considered during surface optimization [31].

Wetting characteristics play a crucial role in saliva-mediated lubrication and contribute directly to reduced friction in orthodontic applications [32]. Poor wettability (high WCA) limits saliva spreading, favoring boundary or dry friction regimes. Improved wettability (low WCA) promotes the formation of continuous saliva films, enabling mixed or hydrodynamic lubrication. Therefore, the enhanced hydrophilicity of SLM-CoCrW surfaces supports more effective saliva-mediated lubrication, which is consistent with the observed reduction in friction coefficients. Overall, the friction results demonstrate that an optimal balance between surface topography and surface chemistry is required to minimize friction. While electropolishing produces the smoothest surfaces, mechanical polishing yields the lowest friction under the present experimental conditions, highlighting the complex interplay between roughness, wettability, and interfacial adhesion at the nanoscale.

**Figure 7:**
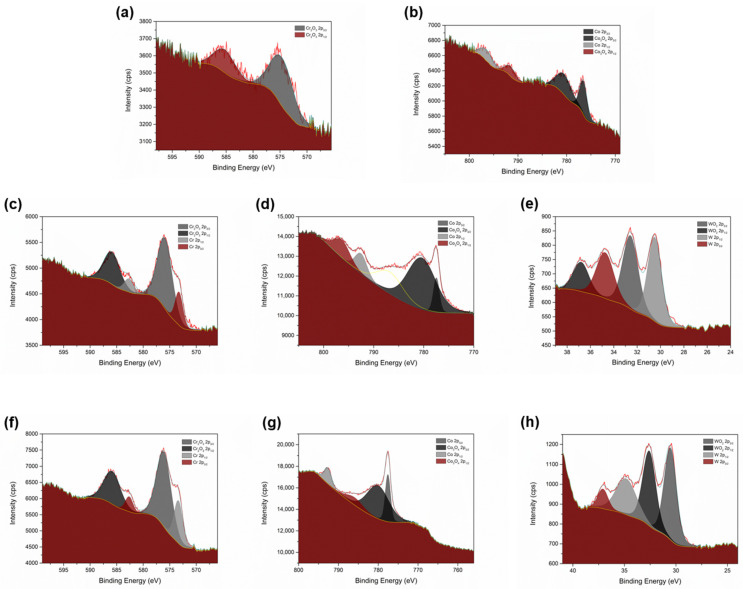
XPS spectra of SLM-CoCrW specimens under different surface conditions. (**a**,**b**) Cr 2p and Co 2p spectra of as-manufactured SLM-CoCrW. (**c**–**e**) Cr 2p, Co 2p, and W 4f spectra of mechanically polished SLM-CoCrW (**f**–**h**) Cr 2p, Co 2p, and W 4f spectra in electropolished SLM-CoCrW.

When the overall friction and WCA analyses are considered, it becomes evident that surface chemistry also plays a decisive role in governing the tribological behavior of orthodontic materials. Changes in surface chemical composition directly affect surface free energy and polarity, which determine wettability and the ability of water or saliva to spread and form stable lubricating films at the contact interface [33]. XPS analysis was conducted to elucidate the effect of surface condition on the chemical composition of SLM-CoCrW specimens in the as-manufactured, mechanically polished, and electropolished states (Figure 7).

The XPS of the as-manufactured SLM-CoCrW specimen already shows that mixed oxides dominate the outermost surface, particularly in the areas of Cr 2p and Co 2p (Figure 7a,b). The detected signal in the Cr 2p spectrum (Figure 7a) mainly correlates with Cr_2_O_3_, implying that Cr is present mainly in an oxidized passive form on the surface. In addition, the Co 2p spectrum (Figure 7b) shows contributions from metallic Co and Co oxide species, revealing a mixed chemical state [34]. The absence of any significant W 4f signal in the as-manufactured condition implies either that W is at a depth below the XPS detection depth, at a very low surface concentration, or is masked by the strong surface oxide chemistry of Cr and Co [35]. In general, the as-manufactured part of the surface shows a native oxide layer that is dominated by Cr and Co oxides. After mechanical polishing, general chemical components obtained are still found, but they are more clearly resolved and indicate a complex mixed surface state. The Cr 2p region is dominated by Cr_2_O_3_, affirming that Cr still controls passivation behavior (Figure 7c). There are, however, minor contributions of metallic Cr, which are better reflected in that the native oxide layer has been partially disrupted, and the fresh metallic regions are exposed locally following abrasive polishing. Co exists in a heterogeneous chemical environment [36]. Moreover, mechanical polishing introduces micro-scratches and residual stresses on the surface, which can be initiation sites for localized corrosion. These surface defects, when expressed in XPS spectra, are often accompanied by increased Co exposure, as indicated by the higher Co/Cr ratio [37]. This phenomenon showcases that it needs to be treated in a way that recovers the protective oxide layer. For instance, unlike the as-manufactured surface, the W 4f spectrum (Figure 7e) becomes visible after mechanical polishing and is dominated by WO_3_-related contributions, indicating that polishing exposes W-containing zones or removes some of the original surface layer, making W chemically detectable in XPS information depth. All in all, the mechanically polished surface illustrates a heterogeneous oxide-covered surface where the oxidized and metallic states are more evident compared to the as-manufactured state.

After electropolishing it is evident that the basic elemental chemistry still remains the same, but the spectral characteristics indicate a more regularized passive surface. The Cr 2p (Figure 7f) spectrum will remain the same, mainly dominated by Cr_2_O_3_, but the oxide contribution seems to become more visible and chemically more definite and, hence, better characterized as a result of the generation of a more continuous Cr-rich passive film [38]. In the Co 2p region (Figure 7g), metallic and oxidized Co states remain, although the oxidized components appear more regular and clearer than those that were mechanically polished, suggesting a more uniform surface chemistry. The W 4f (Figure 7h) spectra are still only dominated by WO_3_, but visible is an oxide doublet (W oxide), confirming that W still remains in the oxidized near-surface region post-electropolishing. Electropolishing seems to reduce chemical heterogeneity and allow the formation of a more uniform Cr-rich passive surface in comparison to mechanical polishing. Cr oxide surfaces are generally considered hydrophilic because they form hydrogen bonds with water molecules [38]; however, the WCA analysis showed a significant decrease in wettability following electropolishing, as demonstrated by the increased WCA. Electropolishing produced a chemically homogeneous Cr-rich oxide film and significantly reduced the surface roughness. AFM measurements confirmed widespread smoothing of surface asperities and a significant reduction in peak-to-valley height differences. The observed wettability changes, therefore, cannot be explained using the Wenzel model alone, as electropolishing changed surface topography and chemistry simultaneously. The enhanced hydrophobicity is probably associated with the development of a chemically uniform, low-energy passive oxide surface and the elimination of high-energy metallic sites and surface heterogeneities left after mechanical polishing. Consequently, water droplets are in contact with a more chemically homogeneous interface that leads to a lower spreading and higher contact angles despite the intrinsically hydrophilic nature of chromium oxide. These results indicate that the wettability of electropolished SLM-CoCrW surfaces is governed by the combination of surface chemistry, oxide homogeneity, and topographical smoothing and not by roughness alone.

When comparing the three surface states, we can clearly see the surface chemistry distinction. The as-manufactured specimen mainly presents a native oxide layer formed of Cr and Co oxides, but W is not present in the outermost layer. In the case of mechanical polishing, this oxide layer itself is maintained, but the surface becomes more chemically heterogeneous due to the high contributions of both metallic and oxidized forms, in addition to detectable W oxide. Following electropolishing, although an oxide coating is still present on the surface, more uniformity in the chemical profile and better passivation are obtained, mainly due to the formation of Cr-rich oxides. Therefore, all three conditions demonstrate passive oxide behavior under control conditions: the as-manufactured form depicts its native oxide composition. The mechanically polished state’s morphology is implied to be more disrupted and diverse. On the other hand, the electropolished state gives them all the most homogeneous and stable state mainly dominated by oxides.

## 4. Conclusions

1. Surface post-processing had a pronounced effect on the surface chemistry of SLM-CoCrW orthodontic materials. In particular, electropolishing promoted the formation of a more uniform Cr-rich passive layer, predominantly composed of Cr_2_O_3_, leading to improved surface chemical homogeneity.
2. The results showed that wettability and frictional behavior were influenced by the combined effects of surface chemistry and surface morphology. Although electropolishing produced the smoothest surface, mechanically polished specimens exhibited lower water contact angles and lower frictional resistance, emphasizing the important contribution of surface topography to overall functional performance.
3. From a clinical perspective, mechanical polishing provided the most favorable balance between surface quality, wettability and frictional behavior. Therefore, it appears to be the most suitable post-processing approach for SLM-CoCrW orthodontic components, particularly in applications where friction control is of primary importance.

## Data Availability

The original contributions presented in the study are included in the article. Further inquiries can be directed to the corresponding author.
